# Purification and characterization of an extracellular esterase with organic solvent tolerance from a halotolerant isolate, *Salimicrobium* sp. LY19

**DOI:** 10.1186/1472-6750-13-108

**Published:** 2013-12-10

**Authors:** Li Xin, Yu Hui-Ying

**Affiliations:** 1 Life Science College, Yuncheng University, 333 Hedong East Street, Yuncheng 044000, China

**Keywords:** Halotolerant, Esterase, Purification, *Salimicrobium*, Organic solvent tolerance

## Abstract

**Background:**

Halotolerant bacteria are excellent sources for selecting novel enzymes. Being intrinsically stable and active under high salinities, enzymes from these prokaryotes have evolved to function optimally under extreme conditions, making them robust biocatalysts with potential applications in harsh industrial processes.

**Results:**

A halotolerant strain LY19 showing lipolytic activity was isolated from saline soil of Yuncheng Salt Lake, China. It was identified as belonging to the genus of *Salimicrobium* by 16S rRNA gene sequence analysis. The extracellular enzyme was purified to homogeneity with molecular mass of 57 kDa by SDS-PAGE. Substrate specificity test revealed that the enzyme preferred short-chain *p*-nitrophenyl esters and exhibited maximum activity towards *p*-nitrophenyl butyrate (*p*-NPB), indicating an esterase activity. The esterase was highly active and stable over broad temperature (20°C-70°C), pH (7.0-10.0) and NaCl concentration (2.5%-25%) ranges, with an optimum at 50°C, pH 7.0 and 5% NaCl. Significant inhibition of the esterase was shown by ethylenediaminetetraacetic acid (EDTA), phenylmethylsulfonyl fluoride (PMSF) and phenylarsine oxide (PAO), which indicated that it was a metalloenzyme with serine and cysteine residues essential for enzyme activity. Moreover, the esterase displayed high activity and stability in the presence of hydrophobic organic solvents with log *P*_ow_ ≥ 0.88 than in the absence of an organic solvent or in the presence of hydrophilic solvents.

**Conclusions:**

Results from the present study indicated the novel extracellular esterase from *Salimicrobium* sp. LY19 exhibited thermostable, alkali-stable, halotolerant and organic solvent-tolerant properties. These features led us to conclude that the esterase may have considerable potential for industrial applications in organic synthesis reactions.

## Background

Esterases (EC 3.1.1.1) represent a family of hydrolases that catalyze the hydrolysis and formation of short-chain fatty acid esters. Because of their broad substrate specificity, highly chemo-, regio-, enantio-selectivity and non-aqueous catalytic properties [1], they have diverse applications in biotechnology which are used as additives in laundry detergents and stereo-specific biocatalysis in pharmaceutical production [2]. However, most industrial processes often require aggressive conditions, which can lead to inactivation of the enzymes. In this sense, novel esterases with better catalytic efficiency and specific properties suitable for special reaction conditions are highly demanded [3]. Extremophiles are bizarre microorganisms that can grow and thrive in extreme environments [4]. Among the extremophiles, halotolerant microorganisms, able to live in saline environments, are good candidates for selecting novel enzymes [5]. Enzymes from these prokaryotes can function optimally under extreme conditions, making them robust biocatalysts with potential applications in harsh industrial processes [6].

The possibility of using enzymes in organic solvents offers numerous advantages when compared to traditional aqueous enzymology, such as high solubility of hydrophobic substrates and reduced water activity which alters the hydrolytic equilibrium and elimination of microbial contamination [7]. Esterases are widely used as biocatalysis due to their ability to catalyze not only the hydrolysis of triacylglycerides in aqueous solutions, but also enantio-selective synthetic reactions in organic media. Therefore, esterase that remains active and stable in the presence of organic solvents might be very useful for biotechnological applications in which such solvents are used. Since salt tends to greatly reduce water activity like organic solvents, enzymes from halotolerant microorganisms may become the choice for biocatalytic processes performed in low water activity environments [8]. So far, numerous organic solvent-tolerant microbial esterases have been reported [9]; however, published studies on the enzymatic behavior of esterases from halotolerant bacteria in non-aqueous media are scarce. Recently, screening of lipolytic activity was carried out among microorganisms from Yuncheng Salt Lake, China. In this work, a halotolerant strain LY19 showing lipolytic activity was isolated and identified. Meanwhile, purification and characterization of its extracellular esterase, especially its activity and stability in the presence of organic solvents, were also reported.

## Results

### Strain identification and production of extracellular esterase

**Figure 1:**
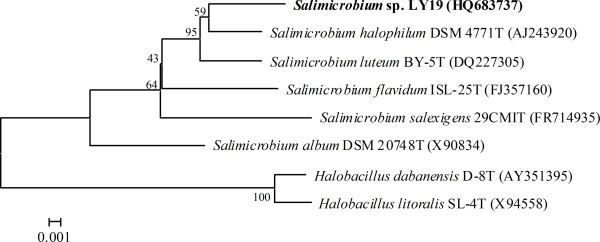
**Phylogenetic tree based on 16S rRNA gene sequence of the isolate LY19 to other members of the genus *Salimicrobium*.** Accession numbers of the sequences used in this study are shown in parentheses after the strain designation. Numbers at nodes are percentage bootstrap values based on 1,000 replications; only values greater than 50% are shown. Bar 0.001 substitutions per nucleotide position.

The strain LY19 is a Gram-positive, rod shaped and aerobic bacterium. Colonies are light yellow on CM agar plate. It is able to grow in media containing 0-30% (w/v) NaCl and grows optimally at 4% (w/v) NaCl. Thus this bacterium can be considered as a halotolerant microorganism [10]. Optimal temperature and pH ranges for bacterial growth are 37°C-39°C and 6–8, respectively. H_2_S production, methyl red and tween-60 hydrolysis are negative, while Voges-Proskauer test, nitrate reduction, catalase and starch hydrolysis are positive. Acid is produced from maltose, sucrose and glucose. Phylogenetic analysis based on 16S rRNA gene sequence comparisons revealed the strain LY19 belonged to the genus of *Salimicrobium* and was most closely related to *Salimicrobium halophilum* DSM 4771 T (98.6% 16S rRNA gene sequence similarity) (Figure 1). Thus, it was tentatively named as *Salimicrobium* sp. LY19.

**Figure 2:**
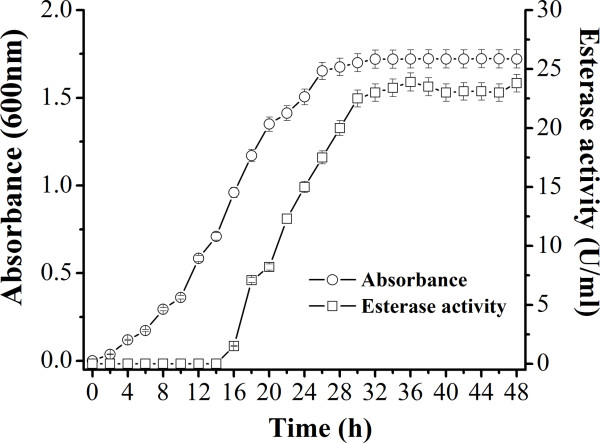
**Bacterial growth and esterase production of stain LY19 in CM broth containing 4% (w/v) NaCl at 37°C.** Results represent the means of three separate experiments.

As shown in Figure 2, the esterase was produced from the middle-exponential phase of bacterial growth (16 h), and reached a maximum level during the stationary phase (34 h). No esterase activity was detected during the early-exponential growth phase.

### Esterase purification

**Figure 3:**
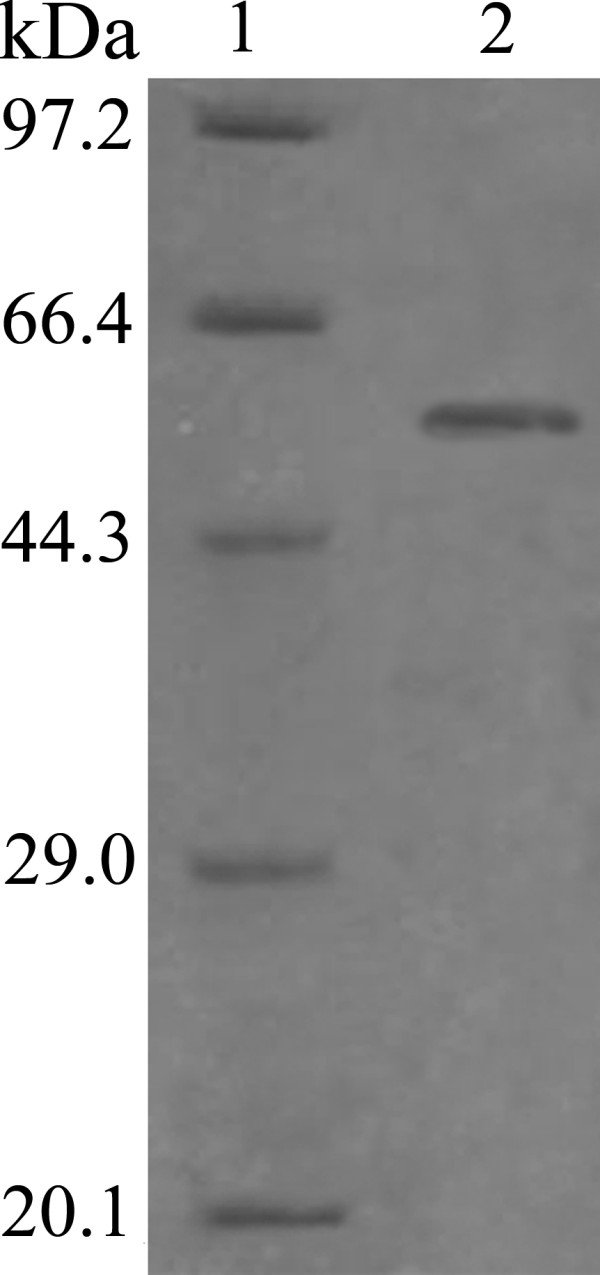
**SDS-PAGE analysis of the purified esterase.** Lane **1**: molecular mass markers; lane **2**: purified esterase.

The extracellular esterase from strain LY19 was well purified by 60% ammonium sulphate precipitation, DEAE-Sepharose anion exchange chromatography and Sephacryl S-100 gel filtration chromatography. It was purified 6.9-fold with recovery of 14.6% recovery and specific activity of 124.2 units/mg protein using *p*-NPB as substrate (Table 1). The purified esterase showed a single protein band on SDS-PAGE with an estimated molecular mass of 57 kDa (Figure 3, lane 2), corresponding with that determined by gel filtration. Together the results suggested that the esterase was a single polypeptide chain.

**Table 1:** **Results of the esterase purification from *Salimicrobium *** **sp. LY19**

**Purification steps**	**Total activity (U)**	**Total protein (mg)**	**Specific activity (U/mg)**	**Purification (fold)**	**Yield (%)**
Crude enzyme	1020	57.1	17.9	1.0	100
60% (NH_4_)_2_SO_4_	725	17.3	41.9	2.3	71.1
DEAE-Cellulose	361	4.4	82.0	4.6	35.4
Sephacryl S-100	149	1.2	124.2	6.9	14.6

### Substrate specificity

**Figure 4:**
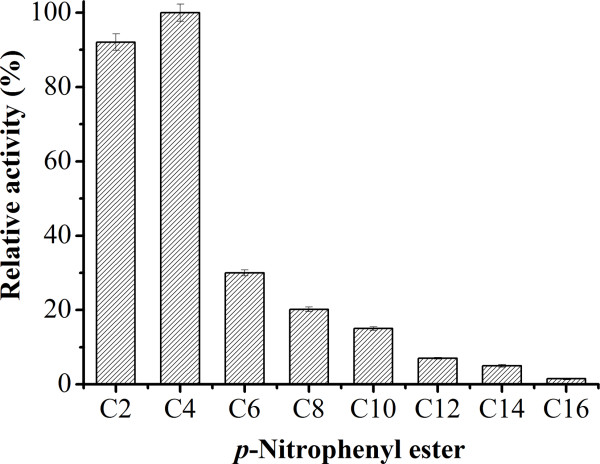
**Substrate specificity of the esterase towards the *p*-NP esters.** Assays were done with 5% NaCl at 50°C and pH 7.0. Results represent the means of three separate experiments.

The substrate specificity of the enzyme was determined using different *p*-NP esters with acyl chain lengths from C2 to C16 (Figure 4). Maximal hydrolytic activity was obtained against *p*-NPB (C4). Enzyme activity declined along with longer chain-length, reaching 30% activity with *p*-NPH (C6), 20.2% with *p*-NPD (C8) and 15% with *p*-NPL (C10), respectively. Little activity was detected against *p*-NP esters with acyl chain lengths longer than C12. Together with these results indicated the enzyme was an esterase for short-chain *p*-NP esters.

### Effect of metal ions and chemical reagents

As shown in Table 2, Ca^2+^ was found to stimulate the esterase activity (138.9%), whereas Hg^2+^ inhibited the enzyme with about 81.6% activity lost. Other metal ions tested had little effects. After incubation with EDTA, only 8.9% of its original activity retained. Meanwhile, PMSF and PAO also showed significant inhibition on the esterase activity. Other enzyme inhibitors, such as β-mercaptoethanol and DEPC, had no obvious effect on the enzyme.

**Table 2:** Effects of metal ions and chemical reagents on esterase activity

**Substances**	**Concentration (mM)**	**Residual activity (% ± SD)**
Control	-	100
Ca^2+^	5	138.9 ± 1.6
Zn^2+^	5	97.6 ± 0.8
Fe^2+^	5	92.7 ± 1.7
Fe^3+^	5	91.8 ± 0.9
Cu^2+^	5	80.8 ± 1.8
Mn^2+^	5	97.5 ± 1.6
Hg^2+^	5	18.4 ± 0.4
Mg^2+^	5	97.7 ± 1.3
EDTA	1	8.9 ± 0.2
PMSF	1	11.1 ± 0.2
DEPC	1	92.1 ± 1.1
PAO	1	8.4 ± 0.3
β-mercaptoethanol	1	91.3 ± 1.5

Residual activity was determined as described in “Methods” and expressed as the percentage of the control value (without any additives). Values are expressed as the averages of three independent experiments ± standard deviations.

### Effects of temperature, pH and NaCl concentration

**Figure 5:**
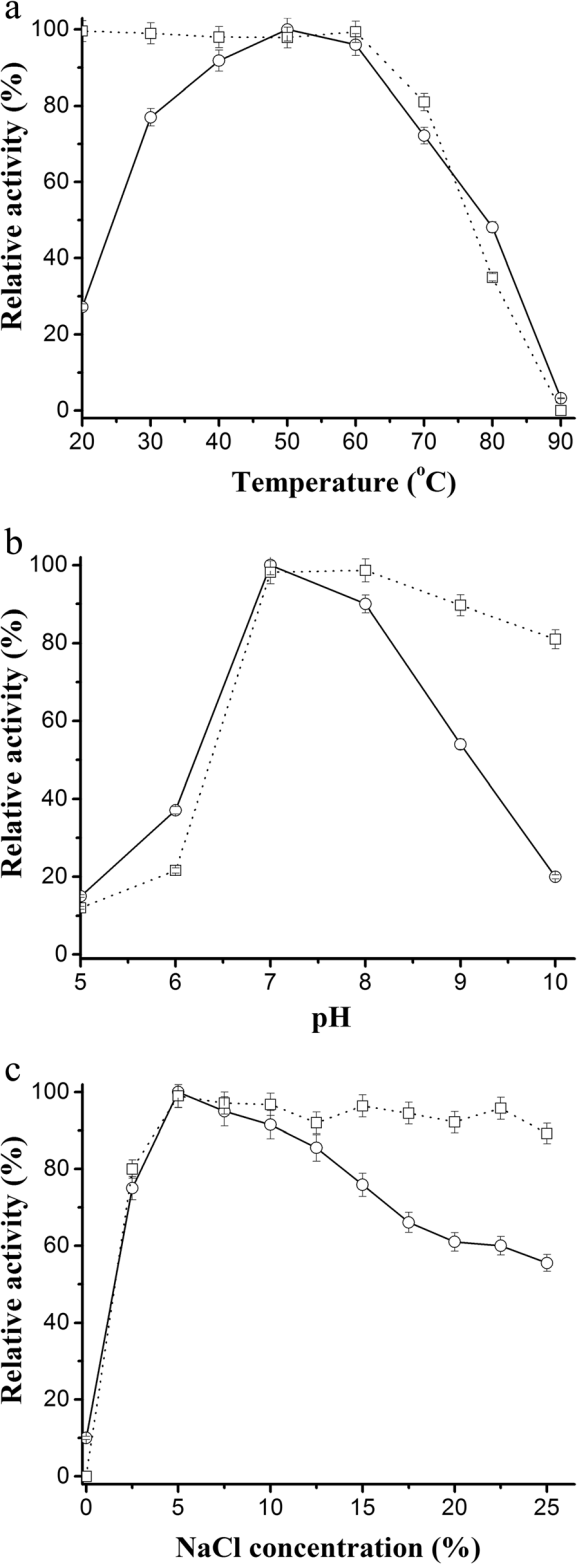
**Effect of temperature (a), pH (b) and NaCl concentration (c) on activity (solid lines) and stability (dotted lines) of the purified esterase.** Relative activity was defined as the percentage of activity detected with respect to the maximum enzyme activity. For determining the stability, the enzyme activity without any treatment was taken as 100%. Data are the average of three independent experiments. See “Methods” for further details.

The temperature profile of esterase activity was shown in Figure 5a. The enzyme displayed optimal activity at 50°C and it retained 48% activity at 80°C. Thermostability test showed the enzyme was highly stable at temperatures below 70°C with more than 80% activity retained. However, after 2-h incubation at 90°C, the enzyme activity was completely lost. Effect of pH on esterase activity and stability was shown in Figure 5b. Optimal pH for the enzyme activity was 7.0. Activity dropped off quickly in more acidic or alkaline conditions, as more than 80% activity lost at pH 5.0 and 10.0. It was highly stable in the pH range of 7.0-10.0 after 2-h incubation. Esterase activity was also measured in the presence of NaCl concentrations ranging from 0 to 25%, and optimal activity was found to be at 5% NaCl (Figure 5c). It was highly stable in the presence of NaCl concentrations from 5% to 25% and retained 80% activity in the presence of 2.5% NaCl. However, little activity was detected in the absence of NaCl.

### Effect of organic solvents

As shown in Table 3, more than 80% activity retained in the presence of glycerol, DMSO, benzene, *n*-hexane or isooctane compared to the control. Isooctane even increased the esterase activity to 119.1%. However, exposure to hydrophilic solvents, such as acetonitrile, ethanol and acetone, enzyme activity and stability were drastically reduced. In addition, the esterase displayed considerable stability in the presence of hydrophobic organic solvents (log *P*_ow_ 0.88-4.7), which showed longer half-lives in these solvents than in the absence of organic solvent (Table 3).

**Table 3:** Activity and stability of the esterase in different organic solvents

**Organic solvents**	**Log *P***_**ow**_^**a**^	**Residual activity (%)**
Control^b^	−	100 (3 d)^c^
Glycerol	−1.76	81.1 (2 d)
DMSO	−1.35	81.4 (1 d)
DMF	−1.0	68.9 (1 d)
Methanol	−0.76	18.4 (<1 h)
Acetonitrile	−0.34	11.9 (<1 h)
Ethanol	−0.3	15.5 (<1 h)
Acetone	−0.24	17.3 (1 d)
1-Butanol	0.88	67.9 (4 d)
Chloroform	1.97	62.8 (4 d)
Benzene	2.13	87.3 (4 d)
Toluene	2.73	64.1 (>5 d)
Cyclohexane	3.3	65.1 (>5 d)
*n*-Hexane	3.5	86.6 (>5 d)
1-Decanol	4.1	73.9 (>5 d)
Isooctane	4.7	119.1 (>5 d)

^a^The log *P*_ow_ is the logarithm of the partition coefficient, *P*, of the solvent between *n*-octanol and water and is used as a quantitative measure of the solvent polarity.

^b^The activity of the purified esterase in the absence of organic solvents was taken as control (100%).

^c^The numbers in brackets are the half-lives of the enzyme in different organic solvents.

## Discussion

In recent years, the ability of extremophiles to grow under harsh conditions makes them very attractive for screening of novel enzymes with unusual properties. In this paper, a halotolerant strain LY19 showing lipolytic activity was isolated and identified as belonging to the genus *Salimicrobium* by 16S rRNA gene sequence analysis (Figure 1). Esterase activity was detected at the mid-exponential phase of bacterial growth and reached a maximum level during the stationary phase. Besides, supplementation of tween-20 as an inducer was required for esterase production. Thus, it was an inducible enzyme secreted into the culture medium. This finding was similar to the lipase from *Penicillium* sp. DS-39 [11]. Molecular mass of the esterase was estimated to be 57 kDa, which was higher than other halophilic esterases: 45 kDa from *Thalassobacillus* sp. strain DF-E4 [12] and 50 kDa from *Haloarcula marismortui*[3]. Substrate specificity test revealed the enzyme was an esterase, as it preferred short-chain *p*-NP esters (C2 and C4) and had very low ability to hydrolyze long-chain esters. Furthermore, lipolytic activity on Rhodamine B agar plates [13] showed it could not hydrolyze olive oil. The esterase from halotolerant bacterium *Pelagibacterium halotolerans* B2T was also reported to show similar substrate specificity [14]. The esterase activity was greatly inhibited by the metal chelator EDTA, indicating hat it was a metalloenzyme. The presence of PMSF (a serine modifier) and PAO (a cysteine modifier) led to the inactivation of the enzyme, which meant that serine and cysteine residues were essential for its catalytic function. Such structural characteristics have not been previously reported for other esterases.

The extracellular esterase can be classified as moderately thermoactive enzyme with optimal activity at 50°C. However, it was worthy noting that the enzyme showed high stability under temperatures below 70°C. After incubation at 80°C for 2 h, about 40% activity still retained. In contrast, other halophilic esterases described previously were inactive under temperatures higher than 70°C [12,15]. Excellent thermostability may favor its application in processes that lead to inactivation of enzymes with increasing temperature. Optimal pH for the esterase was found to be 7.0, which was similar to the intracellular esterase of *Halobacterium* sp. NRC-1 exhibiting its maximum activity at pH 7.5 [15]. The esterase showed good stability in the pH range 7.0-10.0, indicating its alkali-stable property. Similarly, a carboxylesterase from *Thalassobacillus* sp. strain DF-E4 was reported to be active and stable in neutral to alkaline pH range [12]. Alkaline enzymes have received considerable interest because of their tremendous potentiality in industrial processes [16]. Furthermore, the esterase from strain LY19 showed strong tolerance to NaCl. It was highly active and stable in the presence of NaCl concentrations from 2.5 to 25%. This unique property suggested it was a halotolerant enzyme. Similar extreme halotolerance has been observed in other esterases from halophiles [12,17]. Like most halophlic enzymes which were inactive under low salt concentrations [18], the esterase activity reduced drastically in the absence of NaCl, indicating it required salt for maintaining enzyme activity.

High activity and stability of enzyme in organic solvents is an essential prerequisite for applications in organic synthesis [9]. Effect of organic solvents on the esterase from *Salimicrobium* sp. LY19 was shown in Table 3. Significant esterase inactivation in the presence of hydrophilic organic solvents, such as methanol, acetonitrile, ethanol and acetone, was observed, which maybe due to the stripping-off of crucial bound-water monolayer from the enzyme molecule essential for its activity [19]. Although esterases are diverse in their sensitivity to solvents, there is a tendency for hydrophilic solvents to cause more significant enzyme inactivation than hydrophobic solvents [9]. Interestingly, the esterase activity increased greatly in the presence of isooctane. This activation could be explained that organic solvent molecules could interact with hydrophobic amino acid residues present in the lid that covers the catalytic site of the enzyme, thereby maintaining the esterase in its open conformation and conducting to catalyze [20]. Besides, in the presence of hydrophobic organic solvents, the half-lives of the esterase were much longer than in the absence of the solvents or in the presence of hydrophilic solvents. Together these results indicated that, in contrast to the organic solvent stability of some lipases [21], which had no relationship with the polarity of the organic solvents, the stability of the esterase from strain LY9 was probably dependent on the polarity of the solvents, which increased only in non-polar organic solvents with higher log *P*_ow_ values. Similar findings were also observed in some halophilic lipases, which showed high tolerance towards non-polar hydrophobic solvents with significant instability in polar solvents [11,22].

## Conclusions

In the present investigation, a halotolerant strain *Salimicrobium* sp. LY19 producing extracellular esterase was isolated and identified. The esterase was purified to homogeneity with molecular mass of 57 kDa. It was a novel metalloenzyme with serine and cysteine residues essential for enzyme catalysis. Also, considering its thermostable, alkali-stable, halotolerant, and organic solvent-tolerant properties, the esterase might be potentially useful for future applications in biotechnological processes under harsh conditions.

## Methods

### Strain isolation, identification and esterase production

The strain LY19 was isolated from the saline soil of Yuncheng, China. Production of esterase was performed in the complex medium (CM) containing (g/l): casein peptone 7.5; yeast extract 10.0; sodium citrate 3.0; MgSO_4_•7H_2_O 20.0; KCl 2.0; FeSO_4_•7H_2_O 0.01; NaCl 40.0 and pH 7.0. Morphological, physiological and biochemical characteristics of strain LY19 were studied either on CM agar plate (2% agar, w/v) or in CM broth plus 4% NaCl. 16S rRNA gene was amplified using the general bacterial primers 8 F (5′-AGAGTTTGATCCTGGCTCAG-3′) and 1492R (5′-TACCTTGTTACGACTT-3′), and has been deposited to GenBank with the accession number HQ683737. The strain LY19 was deposited at China Center of Industrial Culture Collection with the accession number CICC 10492. The strain LY19 was incubated aerobically in CM broth supplemented with 1% (v/v) tween-20 at 37°C for 48 h with shaking. After centrifugation at 6,000 g for 15 min, cell-free supernatant was used for esterase purification.

### Enzyme purification

The culture supernatant was treated with solid ammonium sulphate to 60% saturation and stirred overnight at 4°C. The precipitate collected by centrifugation was dissolved in buffer A (20 mM Tris–HCl containing 5% NaCl, pH 7.0). After dialysis against buffer A overnight, the sample was applied to a DEAE- Cellulose column (2.5 cm × 30 cm). The column was eluted with a linear gradient of 0–1 M NaCl in Tris–HCl buffer at a flow rate of 0.6 ml/min. Active fractions showing esterase activity were pooled and concentrated by freeze-drying. The resulting concentrate was dissolved in buffer A, and then loaded on a Sephacryl S-100 gel filtration column (1.6 cm × 60 cm). The bound protein was eluted with buffer A at a flow rate of 0.2 ml/min. Active fractions were pooled and used for further characterization. The molecular mass of the purified enzyme was estimated using the same column, which was calibrated previously with bovine serum albumin (BSA) (67 kDa), ovalbumin (43 kDa), bovine carbonic anhydrase (29 kDa) and cytochrome C (12.4 kDa). Blue Dextran was used to determine the void volume of the column. Protein concentration was determined by the method of Bradford [23], using bovine serum albumin as standard. Sodium dodecyl sulfate-polyacrylamide gel electrophoresis (SDS-PAGE) was performed to determine the purity and molecular mass of the esterase on 12% (w/v) polyacrylamide gel [24]. After electrophoresis, the gel was stained with Coomassie Brilliant Blue R-250.

### Enzyme activity assay

The esterase activity was determined using *p*-NPB (*p*-nitrophenyl butyrate) as substrate. 0.4 ml of substrate solution (10 mM dissolved in 2-propanol) was mixed with 3.6 ml of Tris–HCl buffer (20 mM, pH 7.0) containing 5.8% NaCl. The enzymatic assay was initiated by adding the enzyme solution (0.2 ml) to the reaction mixture and incubated at 50°C for 10 min. The amount of *p*-nitrophenol (*p*-NP) released was measured at 410 nm against a blank. One unit (U) of esterase activity was defined as the amount of enzyme liberating 1 μmol of *p*-NP per minute under the assay conditions. The specific activity was expressed as the units of enzyme activity per milligram of protein.

### Substrate specificity

To determine the substrate specificity of the esterase, *p*-nitrophenyl (*p*-NP) esters with different chain lengths (acetate, C2; butyrate, C4; hexanoate, C6; octanoate, C8; decanoate, C10; laurate, C12; myristate, C14; palmitate, C16) were added to the reaction mixture with the final concentration of 1 mM, respectively, and then the released amount of *p*-nitrophenol was measured at 410 nm. Data were expressed as the percentage of the observed maximal activity obtained with *p*-NPB ester (C4).

### Effects of metal ions and chemical reagents

Effects of different metal ions and chemical reagents [ethylenediaminetetraacetic acid (EDTA), phenylmethylsulfonyl fluoride (PMSF), phenylarsine oxide (PAO), diethyl pyrocarbonate (DEPC), β-mercaptoethanol] on the esterase activity were examined by pre-incubating the enzyme with them at 30°C for 1 h, respectively, and then residual activity was determined under the standard assay conditions. Esterase activity in the absence of any additives was taken as 100%.

### Effects of temperature, pH and NaCl concentration on esterase activity and stability

The temperature optimum of the purified esterase was determined under temperatures from 20°C to 90°C. To assess its thermostability, the enzyme was pre-incubated at different temperatures for 2 h and then residual activity was measured using *p*-NPB method as described above. Effect of pH on the esterase activity was measured over a pH range of 5.0-10.0. The buffers (20 mM) used were as follows: sodium acetate (pH 4.0-5.5), sodium phosphate (pH 6.0-7.5), Tris–HCl (pH 8.0-9.0) and glycine-NaOH (pH 9.5-10.0). The pH stability was examined by pre-incubating the esterase under different pH at 50°C for 2 h, and residual activity was measured as described above. Effect of NaCl was tested by measuring the esterase activity in the reaction mixture containing different NaCl concentrations (0-25%). To determine its salt stability, the esterase was pre-incubated in Tris–HCl buffer (20 mM, pH 7.0) containing various NaCl concentrations at 50°C for 2 h. The residual activity was measured using the standard assay.

### Effect of organic solvents on esterase activity and stability

The effect of organic solvents with different Log *P*_ow_ values at 20% (v/v) concentration on the purified esterase was determined by incubating the enzyme solution with different organic solvents at 30°C with shaking, respectively. At different time intervals, aliquots were withdrawn and residual activity was measured under the standard conditions. If residual activity was more than 50% after 5 d, half-life was taken as “>5 d”. While activity was less than 50% after 1 h, half-life was taken as “<1 h”.

## Competing interests

The authors declare that they have no competing interests.

## Authors’ contributions

HYY and XL designed the study. HYY carried out the bulk of the experiments. Both authors have read and approved the final manuscript.
